# Triple negative breast cancer in North of Morocco: clinicopathologic and prognostic features

**DOI:** 10.1186/s12905-016-0346-y

**Published:** 2016-10-22

**Authors:** Touria Derkaoui, Joaira Bakkach, Mohamed Mansouri, Ali Loudiyi, Mohamed Fihri, Fatima Zahra Alaoui, Amina Barakat, Bouchra El Yemlahi, Hassan Bihri, Naima Ghailani Nourouti, Mohcine Bennani Mechita

**Affiliations:** 1 Human Genomic Research Laboratory, Faculty of Sciences and Techniques of Tangier, University Abdelmalek Essaâdi, Tangier, Morocco; 2 Oncology Clinic Al Amal of Tangier, Tangier, Morocco; 3 Mathematics and Applications Laboratory, Faculty of Sciences and Techniques of Tangier, University Abdelmalek Essaâdi, Tangier, Morocco

**Keywords:** Triple negative breast cancer, BRCA1, BRCA2, Prognostic, Clinicopathologic, Survival analysis

## Abstract

**Background:**

Triple Negative Breast Cancer (TNBC) is defined by a lack of estrogen and progesterone receptor gene expression and by the absence of overexpression on HER2. It is associated to a poor prognosis. We propose to analyze the clinicopathologic and prognostic characteristics of this breast cancer subtype in a Mediterranean population originated or resident in the North of Morocco.

**Methods:**

We conducted a retrospective study of 279 patients diagnosed with breast cancer between January 2010 and January 2015. Clinicopathologic and prognostic features have been analyzed. Disease-Free Survival (DFS) and Overall Survival (OS) have been estimated.

**Results:**

Of all cases, forty-nine (17.6 %) were identified as having triple negative breast cancer with a median age of 46 years. The average tumor size was 3.6 cm. The majority of patients have had invasive ductal carcinoma (91.8 %) and 40.4 % of them were grade III SBR. Nodal metastasis was detected in 38.9 % of the patients and vascular invasion was found in 36.6 % of them. About half of the patients had an early disease (53.1 %) and 46.9 % were diagnosed at an advanced stage. Patients with operable tumors (61.2 %) underwent primary surgery and adjuvant chemotherapy. Patients with no operable tumors (26.5 %) received neoadjuvant chemotherapy followed by surgery, and patients with metastatic disease (12.2 %) were treated by palliative chemotherapy. DFS and OS at 5 years were respectively 83.7 and 71.4 %. Among 49, twelve had recurrences, found either when diagnosing them or after a follow-up. Local relapse was 6.1 %. Lung and liver metastases accounted consecutively for 8.2 and 10.2 %. Bone metastases were found in 4.1 % and brain metastases in 2.1 % of the cases.

**Conclusion:**

Our results are in accordance with literature data, particularly what concerning young age and poor prognosis among TNBC phenotype. Therefore, the identification of BRCA mutations in our population seems to be essential in order to better adapt management options for this aggressive form of breast cancer.

## Background

Breast cancer is a heterogeneous disease encompassing various clinical, morphological and molecular distinct subtypes. In the early 2000s, through RNA expression profile analysis of breast cancer, Perou and Sorlie have identified five main molecular subtypes: Luminal A, Luminal B, ERBB2, Basal-Like (BL) and Normal-Like (NL), bringing out a new classification of this disease and identifying groups with distinct biology and prognosis [1].

In clinical practice, BL tumors are often treated as triple negative breast cancers (TNBCs), tumors which are defined using immunohistochemical methods. TNBCs are characterized by lack of expression of hormone receptors (HR, Estrogen ER and Progesterone PR) and human epidermal growth factor receptor 2 (HER2). This breast cancer phenotype accounts for approximately 17 % in most published series (10∼20 %) [2–4].

TNBC is over-represented among non-menopausal African descent women and is characterized by a high BRCA mutation rate [2, 5–8]. Patients with TNBC are more likely to experience relapse within the first 3 years and are at high risk of death in the first 5 years following diagnosis. Furthermore, TNBC exhibits a high frequency of metastasis compared to other breast cancer phenotypes with distinct metastatic patterns, particularly in visceral and cerebral sites [3, 9–12].

We present here the first study aiming to analyze clinicopathologic and prognostic features of triple negative breast cancer in a Mediterranean population originated or resident in the North of Morocco.

## Methods

### Patients

We describe a retrospective study of breast cancer in a Mediterranean population originated or resident in the North of Morocco. This population represent 10.5 % of the total Moroccan population. After reviewing data of the Oncology Clinic Al Amal of Tangier, a total of 279 breast cancer cases was registered between January 2010 and January 2015. In this work only patients diagnosed with TNBC were recruited.

### Methods

Estrogen and progesterone receptors were evaluated on fixed and paraffin-embedded tissue using immunohistochemical methods. The threshold set out for HR negativity was a lack (<1 % positivity) of any ER and PR immunoreactivity. HER2 was considered negative if immunohistochemical stains were reported 0, 1^+^ or if HER2 FISH showed no gene amplification.

All tumors were classified following histological classification (WHO 2003). Histological grading was performed using Scarff-Bloom-Richardson classification (SBR) as modified by Elston-Ellis (mSBR). Tumors were classified according to the TNM system 2010 and regrouped into American Joint Committee on Cancer stages (AJCC 2010).

Disease-Free Survival (DFS) and Overall Survival (OS) were assessed using Kaplan-Meier analysis [13]. DFS was determined from the time of diagnosis to the date of tumor relapse or last follow-up. OS was defined as the time between date of diagnosis and death or last follow-up. Survival according to AJCC stages was also estimated, early (I, II) and advanced (III, IV) stages. The Log-Rank test was used to examine the statistical significance of the differences observed between the two groups early and advanced. This test was performed at a 5 % level.

Participants provide written informed consent. Ethic approval for the study was obtained through the Ethics Committee for Biomedical Research in the Faculty of Medicine and Pharmacy of Rabat (CERB).

## Results

Among 279 patients diagnosed with breast cancer, forty-nine (17.6 %) were identified as triple negative. The median age at diagnosis was 46 and was ranging from 28 to 90 years. Approximately two thirds of patients (63.6 %) were non-menopausal and 24.4 % had a family history of breast cancer. The main ethnic groups of our population are Arabs (69.4 %) and Amazigh (26.6 %). Europeans and sub-Saharan represent only 2 % each.

The average tumor size was 3.6 cm. The overwhelming majority of patients (91.8 %) had an infiltrating ductal carcinoma, and 8.2 % of cases had infiltrating lobular carcinoma. High-grade tumors represented 40.4 % of cases, the intermediate 55.3 % and low-grade was only 4.3 %. Regarding lymph node involvement, 38.9 % of patients had positive lymph nodes at initial diagnosis and a lymphovascular invasion was found in 36.6 % of cases. Patients with stage I accounted for 12.2 %, stage II (40.8 %), stage III (34.7 %) and 12.2 % had metastatic disease (stage IV)(Table 1).

**Table 1:** Clinicopathologic characteristics of the population study

Variable	Frequency
TNBC	17.6 %
Median age at diagnosis	46 ans
Ethnicity	
Arab	69.4 %
Amazigh	26.6 %
Sub-Saharan	2 %
European	2 %
Menopause	
Yes	36.4 %
No	63.6 %
Breast cancer family history	
Present	24.4 %
Absent	75.6 %
Tumor size	
T1	10.2 %
T2	73.5 %
T3	12.2 %
T4	4.1 %
Histological type	
Invasive ductalcarcinoma	91.8 %
Invasive lobularcarcinoma	8.2 %
SBR grading	
I	4.3 %
II	55.3 %
III	40.4 %
Lymphovascular invasion	
Present	36.6 %
Absent	63.4 %
Lymph node status	
N0	61.1 %
N1	25 %
N2	5.6 %
N3	8.3 %
Metastasisat initial diagnosis	
M0	87.8 %
M1	12.2 %
TNM stage	
I	12.2 %
II	40.8 %
III	34.7 %
IV	12.2 %
Distant metastasis	
M0	86 %
M1	14 %
Recurrence sites	
Local	6.1 %
Bone	4.1 %
Liver	10.2 %
Lung	8.2 %
Brain	2.1 %

Patients with operable tumors (T1–T2–T3, N0–N1) which represent 61.2 % in our series, underwent primary surgery and adjuvant chemotherapy. Patients with no operable tumors (T4, N2–N3) (26.5 %) received neoadjuvant chemotherapy followed by surgery. Metastatic cancers (12.2 %) received palliative chemotherapy. Breast-conserving surgery was performed in 31.6 % of cases, either immediately or after neoadjuvant chemotherapy (Table 2).

**Table 2:** Treatment modalities for all patients

Surgery	
Radical surgery	68.4 %
Conservingsurgery	31.6 %
Chemotherapy	
Adjuvant	61 %
Neoadjuvant	26.8 %
Palliative	12.2 %
Radiotherapy	
Yes	70.7 %
No	29.3 %

The majority of patients received radiotherapy (70.7 %). Patients who underwent breast-conserving surgery, breast irradiation was systematically performed in negative lymph nodes cases (77.8 %). For patients with lymph nodes metastasis, nodal irradiation was constantly indicated (22.2 %). About patients who underwent radical surgery, chest wall irradiation was applied in the absence of lymph node invasion, at 64.7 % considering the TN status as a poor prognostic factor indicating radiotherapy. Chest wall and nodal complete irradiation was performed for patients who have positive lymph nodes (35.3 %).

**Fig. 1:**
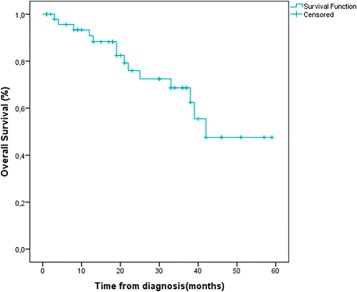
Overall survival at 5 years for all patients

**Fig. 2:**
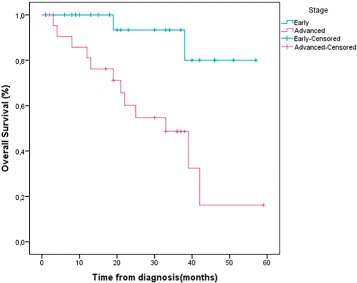
Survival for all patients according to AJCC staging

**Fig. 3:**
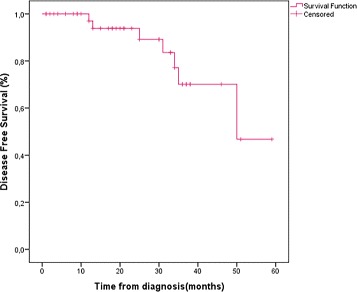
Disease-free survival at 5 years for non-metastatic patients

Five years disease-free survival for patients with localized disease was 83.7 %. The overall 5 years survival for all patients was 71.4 % (Table 3, Figs. 1 and 2). Patients diagnosed in an advanced stage had shorter survival rate than those diagnosed at early stage (47.8 %*versus* 92.3 % consecutively, with *p* *v* *a* *l* *u* *e*=0.002<0.05 (Table 3, Fig. 3)).

**Table 3:** Outcome of the triple negative group

Kaplan-Meier survival result	
Disease free survival	83.7 %
Overall survival at 5 years	71.4 %
Early	92.3 %
Advanced	47.8 %

Among 49 patients, 12 had recurrence either at diagnosis or after follow-up. In this group, local recurrence was 6.1 %. The distribution of metastatic sites was as following: lung (8.2 %), liver (10.2 %), bone (4.1 %) and brain (2.1 %)(Table 1).

## Discussion

Triple negative breast cancer frequency reported in the present work (17.6 %) is consistent with national [14, 15] and international data [2–4]. This frequency varies according to race/ethnicity and positivity threshold set out for HR and HER2 expression [2, 16, 17]. Morocco is a country of northwestern Africa whose population is characterized by ethnic diversity. Arab and Amazigh present the majority of this population that contain also other groups as Jewish, European and sub-Saharan. Ethnic composition of our series bears a great resemblance to that of the whole country. Arab and Amazigh are predominant (96 %). As our case-series is limited, we can not give any conclusion about TNBC distribution. Furthermore, to the best of our knowledge, there is no data about TNBC frequency among these ethnic groups.

TNBC occurs more frequently at a younger age, often before menopause compared with other breast cancer subtypes [3]. In the current study, the median age was 46 years and the majority of our patients were non-menopausal (63.6 %). Early age of onset for breast cancer can reflect a genetic predisposition in particular mutations of BRCA1 and BRCA2 genes. A strong correlation has been described between the BRCA pathway and triple negative breast cancers. The prevalence of germline mutations in BRCA genes among triple negative patients varies between 9 and 20 % [18–21].

Although somatic mutations in BRCA1 gene represent about 40 %, there is a highly dysfunction in BRCA1 within tumor cells by other mechanisms, notably epigenetic modifications like gene methylation [20, 22–24].

Inactivation of BRCA1 gene, which is commonly described between BRCA1 mutations carriers and TNBC, is responsible for a dysfunction of DNA repair. Hence, this pathway could be exploited therapeutically by poly (ADP-ribose) polymerase inhibitors (PARP), repair enzymes for single strand breaks in DNA, which is the only repair pathway available currently [25–27]. Besides, this similarity has nowadays important implications in clinical management by genetic testing for germline BRCA1 mutations, notably in young premenopausal woman, even without a family history. In the present study, 24.4 % of patients had a familial history of breast cancer. According to the Guidelines of National Comprehensive Cancer Network, TNBC has become a supplementary individual criterion for genetic testing. A genetic study of BRCA genes involving all patients included in this work is ongoing.

Clinically, TN tumors are often large [3]. In this study, the mean diameter was 3.6 cm. This could be related to rapid growth of this subtype of tumors and to its relatively high incidence in young patients who present difficulties in mammography detection.

The great majority of TN tumors are invasive ductal carcinomas (Invasive Breast Carcinomas of No Special Type (NST), WHO classification 2012) with high histologic grade. Interestingly, some TNBCs comprise special histological subtypes with a favorable prognosis, such as adenoid cystic carcinoma (called cylindrome) and Juvenile secretory carcinoma [28, 29]. None of these histological subtypes was reported in the present study.

Traditionally, as tumor size increases, the rate of lymph node involvement increases. This correlation was not observed in our group study. The majority of tumors (85.7 %) had a diameter superior than 2 cm, paradoxically, 61.1 % of patients were negative for nodal involvement. These results are consistent with those found in literature data [3, 30].

Triple negative breast tumors have demonstrated high chemosensitivity. Such tumors responded in the neoadjuvant setting and have showed complete pathologic response (pCR) generally higher than non-TNBCs. This is the “paradox of TNBC” [31–34].

For patients with inoperable disease (large size, inflammatory tumor, etc.), neoadjuvant chemotherapy provides a means to improve further surgery. Moreover, it gives the opportunity to propose later, depending on the quality and the terms of response, a breast-conserving surgery improving thus the cosmetic and preserving more women’s body image.

Regarding patients with operable disease, neoadjuvant chemotherapy allows in one hand to test in vivo the treatment effectiveness and to predict pCR rate, which is a good prognostic factor. On the other hand, it provides necessary time to genetic testing for BRCA mutations especially among women aged under 50. This allows guiding management process for mutation carriers. Radical surgery would be recommended. Preventive measures, such as prophylactic surgery could be also discussed.

In the present study, the rates of advanced and metastatic cancers are higher (46.9 and 12.2 % respectively). This is due mainly to lack of early detection in our population. Therefore, implementation of a national organized screening program for breast cancer is essential.

The correlation between TN status and increased risk of local recurrence remains controversial. Some studies did not show an increased risk. Solin et al. [35] revealed that local recurrence rate at 8 years was 8 % compared to 4 % in non-TNBC. Freedman et al. [36] have reported, by analyzing three groups HR+, HER2 and TN, that the local recurrence rates at 5 years were similar in the three groups (2.3, 4.6 and 3.2 % respectively). However in Nguyen et al. [37] study, the rate of local recurrence at 5 years was significantly higher in the TN group compared with Luminal A and B cancers (7.1 % *versus* 0.8 % and 1.5 %). In the present study, the local recurrence rate was 6.1 %.

The TN group presents a significant risk of recurrence within the first 3 years and death during the 5 years following initial diagnosis [3, 9], the risk of relapse declines rapidly thereafter. After 8 years of follow up, recurrence is exceptional [3].

Metastatic progression is characterized by predominance of visceral sites (notably liver and brain) and lower incidence of bone metastases [9–12]. In the current study, we showed a high rate of visceral metastases including liver (10.2 %) and lungs (8.2 %), but a lower rate of brain metastases (2.1 %). Bone metastases were found in 4.1 % of cases.

Prognosis for TN cancers remains pejorative compared to other subtypes, whether in terms of disease-free survival or overall survival [2, 3, 38, 39]. DFS and OS at 5 years were respectively 83.7 and 71.4 %. Most recurrences were recorded in the first 2 to 3 years following diagnosis. These results are in accordance with literature data [3, 9].

The TNBCs diagnosed at an early stage presented a five-year survival rate which is strikingly higher (92.3 %) than advanced TNBCs (47.8 %). Early detection is therefore one of the most powerful strategies in reducing cancer mortality and improving chances of recovery.

## Conclusion

The characteristics of TNBC among our population study are consistent with international literature data, especially concerning young age at diagnosis, poor prognosis and pejorative survival. Further research studies for determining BRCA abnormalities will lead to a better understanding the genetic profile of our population.

The identification of germline BRCA mutations in triple negative breast tumors seems essential to adapt the therapeutic strategy and to ensure adequate clinical management for mutation carriers and their relatives.

At the somatic level, characterization of molecular defects in triple negative tumors mainly BRCA and p53, are promising for new targeted therapeutic approaches. This is the case of PARP inhibitors that target the inactivation of the enzyme poly (ADP-ribose) polymerase, the only pathway so far available for DNA breaks repair.

## Abbreviations

DFS: Disease-free survival
ER: Estrogen receptor
FISH: Fluorescence in situ hybridization
HER2: Human epidermal growth factor receptor 2
HR: Hormone receptors
NST: No special type
OS: Overall survival
PARP: Poly (ADP-ribose) polymerase
pCR: Complete pathologic response
PR: Progesterone receptor
SBR: Scarff-Bloom-Richardson
TNBC: Triple negative breast cancer
WHO: World Health Organization
